# Estimated Rates of Incident and Persistent Chronic Pain Among US Adults, 2019-2020

**DOI:** 10.1001/jamanetworkopen.2023.13563

**Published:** 2023-05-16

**Authors:** Richard L. Nahin, Termeh Feinberg, Flavia P. Kapos, Gregory W. Terman

**Affiliations:** 1 National Center for Complementary and Integrative Health, National Institutes of Health, Bethesda, Maryland; 2 Kelly Government Solutions, Rockville, Maryland; 3 Center for Child Health, Behavior and Development, Seattle Children’s Research Institute, Seattle, Washington; 4 Department of Anesthesiology and Pain Medicine, University of Washington, Seattle

## Abstract

**Importance:**

Chronic pain risk and prognosis estimates are needed to inform effective interventions.

**Objective:**

To estimate rates of chronic pain and high-impact chronic pain (HICP) incidence and persistence in US adults across demographic groups.

**Design, Setting, and Participants:**

This cohort study examined a nationally representative cohort with 1 year of follow-up (mean [SD], 1.3 [0.3] years). Data from the 2019-2020 National Health Interview Survey (NHIS) Longitudinal Cohort were used to assess the incidence rates of chronic pain across demographic groups. The cohort was created using random cluster probability sampling of noninstitutionalized civilian US adults 18 years or older in 2019. Of 21 161 baseline participants in the 2019 NHIS who were randomly chosen for follow-up, 1746 were excluded due to proxy response(s) or lack of contact information, and 334 were deceased or institutionalized. Of the 19 081 remaining, the final analytic sample of 10 415 adults also participated in the 2020 NHIS. Data were analyzed from January 2022 to March 2023.

**Exposures:**

Self-reported baseline sex, race, ethnicity, age, and college attainment.

**Main Outcomes and Measures:**

Primary outcomes were the incidence rates of chronic pain and HICP, and secondary outcomes were the demographic characteristics and rates across demographic groups. A validated measure of pain status (“In the past 3 months, how often did you have pain? Would you say never, some days, most days, or every day?”) yielded 3 discrete categories each year: pain free, nonchronic pain, or chronic pain (pain “most days” or “every day”). Chronic pain present in both survey years was considered persistent; HICP was defined as chronic pain that limited life or work activities on most days or every day. Rates were reported per 1000 person-years (PY) of follow-up, and age standardized based on the 2010 US adult population.

**Results:**

Among 10 415 participants included in the analytic sample, 51.7% (95% CI, 50.3%-53.1%) were female, 54.0% (95% CI, 52.4%-55.5%) were aged 18 to 49 years, 72.6% (95% CI, 70.7%-74.6%) were White, 84.5% (95% CI, 81.6%-85.3%) were non-Hispanic or non-Latino, and 70.5% (95% CI, 69.1%-71.9%) were not college graduates. Among pain-free adults in 2019, incidence rates of chronic pain and HICP in 2020 were 52.4 (95% CI, 44.9-59.9) and 12.0 (95% CI, 8.2-15.8) cases per 1000 PY, respectively. The rates of persistent chronic pain and persistent HICP in 2020 were 462.0 (95% CI, 439.7-484.3) and 361.2 (95% CI, 265.6-456.8) cases per 1000 PY, respectively.

**Conclusions and Relevance:**

In this cohort study, the incidence of chronic pain was high compared with other chronic diseases. These results emphasize the high disease burden of chronic pain in the US adult population and the need for early management of pain before it becomes chronic.

## Key Points

**Question:**

What are the rates of chronic pain (pain “most days” or “every day”) and high-impact chronic pain (chronic pain that limits life or work activities on most days or every day) incidence and persistence in US adults?

**Findings:**

In this cohort study of 10 415 adult participants in the National Health Interview Survey 2019-2020 Longitudinal Cohort, the incidence rates of chronic pain and high-impact chronic pain in 2020 were 52.4 cases per 1000 person-years (PY) and 12.0 cases per 1000 PY, respectively. Among adults with baseline chronic pain, the rate of persistent chronic pain was 462.0 cases per 1000 PY.

**Meaning:**

These longitudinal data emphasize the high disease burden of chronic pain in the US adult population and the need for early management of pain.

## Teaser

This cohort study uses data from the 2019-2020 National Health Interview Survey Longitudinal Cohort to assess rates of incident and persistent chronic pain and high-impact chronic pain among US adults across demographic groups.

## Introduction

Epidemiological research on chronic pain (pain lasting ≥3 months) and high-impact chronic pain (HICP) (chronic pain associated with substantial restrictions in life activities, including work, social, and self-care activities) in the US has increased substantially since the release of the Institute of Medicine (currently the National Academy of Medicine) report on pain in 2011^1^ and the Department of Health and Human Services National Pain Strategy (NPS) in 2016.^2^ These documents emphasized the need for epidemiological studies of pain in the US population, particularly in subpopulations that may be susceptible to the underreporting and/or undermanagement of pain. Since these publications, numerous nationally representative studies of US adults have described chronic pain and HICP prevalence, which varied depending on the operationalization of chronic pain and the survey year.^3,4,5,6,7^ Far less is understood regarding the incidence of chronic pain in US adults because few longitudinal population-based studies contain high-quality information on pain. Current knowledge of chronic pain incidence in the US is limited to specific communities or groups or health care professionals^8,9,10,11,12,13,14,15,16^ and/or specific age ranges^8,9,11,12,13,15,17^ and/or specific pain locations or conditions^8,9,10,11,12,13,15,16,17,18,19,20^ and/or claims data or medical records.^10,17,19^ Of these, a minority of studies^10,12,16,17^ have presented incidence rates (IRs), defined as the number of new cases of chronic pain per person per year (ie, person-years [PY]) of follow-up among persons without chronic pain at baseline. Compared with cumulative incidence proportions, IRs more adequately account for the time at risk of developing an outcome that may occur in study participants during follow-up. We have not found any studies to date examining chronic pain incidence in a nationally representative sample of all adults.

In addition to identifying the IRs of pain in all adults, we used data from the 2019-2020 National Health Interview Survey (NHIS) Longitudinal Cohort (NHIS-LC) to determine the IRs of chronic pain across demographic groups to refine our understanding of populations with an increased risk of chronic pain, a goal consistent with the US Centers for Disease Control and Prevention Healthy People 2030 pain objectives.^21^ This study responded directly to the Institute of Medicine and NPS calls for improved national surveillance of chronic pain incidence to aid in the identification of subpopulations for whom early interventions may potentially prevent chronic pain onset and related disability.

## Methods

### Study Population

All aspects of data collected for both periods (2019 and 2020) were approved by the National Center for Health Statistics (NCHS) Research Ethics Review Board. Verbal informed consent was obtained from all respondents. On August 19, 2022, National Institutes of Health Institutional Review Board Panel 1 authorized an exemption from review because this cohort study used publicly available deidentified data. This study followed the Strengthening the Reporting of Observational Studies in Epidemiology (STROBE) guideline for cohort studies.^22^

The NHIS is a nationally representative annual survey of the noninstitutionalized US civilian population from 50 states and the District of Columbia.^23^ The NHIS is conducted by the NCHS of the Centers for Disease Control and Prevention and uses a multistage clustered sample design, which oversamples Asian, Black, and Hispanic populations. Applying NCHS-derived sampling weights allows an accurate extrapolation of findings to the noninstitutionalized US civilian population.^23^

Of the 31 997 NHIS participants in 2019, 21 161 were randomly chosen by the NCHS for possible inclusion in the NHIS-LC.^23^ Of those, 1746 participants were excluded due to proxy responses or a lack of contact information, whereas 334 were subsequently deceased or resided in an institutional setting (eFigure in Supplement 1). Of the remaining 19 081 participants, 10 415 agreed to participate in 2020 and were included in this analysis. Primary outcomes were the IRs of chronic pain and HICP, and secondary outcomes were the demographic characteristics and rates of chronic pain and HICP across demographic groups. Data were analyzed from January 2022 to March 2023.

### Operational Definitions of Chronic Pain and HICP 

For the NHIS years 2019 to 2020, adults in the sample were asked 2 questions about pain suggested by the NPS as a validated measure of pain status^2^ and modified by the NCHS: (1) “In the past 3 months, how often did you have pain? Would you say never, some days, most days, or every day?” and (2) “Over the past 3 months, how often did pain limit your life or work activities? Would you say never, some days, most days, or every day?” Chronic pain was defined as pain on most days or every day during the past 3 months, as recommended by the NCHS.^7^ High-impact chronic pain was defined as chronic pain that limited life or work activities on most days or every day during the past 3 months.^7^ Initial national prevalence estimates using these definitions have been published previously.^5,6,7^

Incidence is defined as a new health condition (such as chronic pain) that occurs in a population that is both previously free of the condition and at risk for experiencing it. In the present study, incidence estimates were based on the 3776 individuals who reported being previously pain free in 2019 but having pain in 2020. No attempt was made to impute data for the small percentage of individuals (141 [1.4%]) without data on pain status in 2020. For demographic characteristics, missing data were categorized as unknown. Participants with chronic pain during both periods were considered to have persistent chronic pain.

### Statistical Analysis

We used absolute standard difference values to compare the baseline characteristics for the complete analytic sample (ie, all 10 415 participants in the NHIS-LC) with those 21 582 participants in the 2019 NHIS who were not included in the NHIS-LC (ie, those not randomized for invitation, randomized but found ineligible, and randomized but declined participation) (Table 1).

**Table 1.** Baseline Characteristics of Participants in the 2019 NHIS by Enrollment Status in the 2019-2020 NHIS Longitudinal Cohort

Characteristic	Enrolled in 2019 (longitudinal weights)			Not enrolled in 2019 (sample adult weights)			Standardized difference scores	
	Raw frequency (n = 10 415)	Weighted frequency, thousands (n = 250 917)	Weighted % (95% CI) (n = 100)	Raw frequency (n = 21 582)	Weighted frequency, thousands (n = 175 427)	Weighted % (95% CI) (n = 100)	Unweighted	Weighted
Age group, y								
18-49	4166	135 420	54.0 (52.4-55.5)	10 142	98 222	56.0 (55.0-56.9)	0.142	0.040
≥50	6249	115 497	46.0 (44.5-47.6)	11 440	77 205	44.0 (43.1-45.0)	0.142	0.040
Sex								
Male	4790	121 160	48.3 (46.9-49.7)	9943	84 644	48.3 (47.4-49.1)	0.002	0
Female	5624	129 741	51.7 (50.3-53.1)	11 637	90 770	51.7 (50.9-52.6)	0.002	0
Unknown	1	15	UR	2	13	UR	NA	NA
Race								
American Indian or Alaska Native	183	4629	1.8 (1.0-2.7)	373	3173	1.8 (1.4-2.3)	0.008	0
Asian	518	14 443	5.8 (4.9-6.6)	1154	10 632	6.1 (5.5-6.7)	0.014	0.009
Black or African American	1025	30 618	12.2 (10.8-13.6)	2567	22 613	12.9 (11.9-13.9)	0.068	0.022
White	8168	182 205	72.6 (70.7-74.6)	16 081	124 048	70.7 (69.3-72.1)	0.092	0.043
Other^a^	106	2968	1.2 (0.9-1.5)	267	2431	1.4 (1.1-1.6)	0.019	0.009
Unknown	415	16 054	6.4 (5.4-7.4)	1140	12 531	7.1 (6.4-7.9)	0.062	0.028
Hispanic or Latino ethnicity								
Chicano, Mexican, or Mexican American	672	25 263	10.1 (8.5-11.6)	1684	17 922	10.2 (9.1-11.4)	0.050	<0.001
Non-Hispanic or non-Latino	9263	209 417	83.5 (81.6-85.3)	18 588	144 750	82.5 (81.1-83.9)	0.085	0.027
Other Hispanic or Latino^b^	470	16 124	6.4 (5.3-7.5)	1265	12 349	7.0 (6.2-7.8)	0.063	0.024
Unknown	10	112	UR	45	407	0.2 (0.1-0.3)	NA	NA
College attainment								
No degree	6148	176 870	70.5 (69.1-71.9)	14 393	127 672	72.8 (71.8-73.8)	0.160	0.040
Degree	4228	72 259	28.8 (27.4-30.2)	7049	46 347	26.4 (25.4-27.4)	0.164	0.056
Unknown	39	1787	0.7 (0.4-1.0)	140	1409	0.8 (0.6-1.0)	0.028	0.012
Pain status in 2019								
No pain	3776	101 143	40.3 (38.8-41.8)	8266	71 423	40.7 (39.7-41.7)	0.041	0.008
Nonchronic pain	4185	97 507	38.9 (37.5-40.2)	7893	63 671	36.3 (35.5-37.1)	0.074	0.054
Chronic pain	2446	52 078	20.8 (19.6-21.9)	4738	34 013	19.4 (18.7-20.1)	0.036	0.035
Unknown	8	187	0.1 (0-0.2)	685	6320	3.6 (3.2-4.0)	0.245	0.262

Abbreviations: NA, not applicable; NHIS, National Health Interview Survey; UR, unreliable (data did not meet National Center for Health Statistics standards for reliability in national surveys^24^).

^a^ Other race includes self-reported Native Hawaiian, Pacific Islander, more than 1 race, and “some other race.”

^b^ Other Hispanic or Latino ethnicity includes self-reported Central American, Cuban, Dominican, Puerto Rican, South American, and other Hispanic, Latino, or Spanish.

We calculated the proportions of participants reporting no pain, nonchronic pain, and chronic pain in 2020, stratified by pain status at baseline. Crude and age-standardized rates and 95% CIs for chronic pain and HICP per 1000 PY were calculated, as were rates by demographic characteristics previously associated with chronic pain: age, sex, race, Hispanic or Latino ethnicity, and college graduation status (college graduate vs not college graduate), all of which were self-reported in this cohort (Table 1). Age was coded as 18 to 49 years and 50 years or older, with age 49 years being the approximate median of the sample. Preliminary analyses revealed that quartile and tertile distributions of age yielded unreliable estimates^19^ of chronic pain and/or HICP incidence, as did the categorization of educational attainment into 3 groups (<high school, high school graduate, and college graduate). In the NHIS data set, self-reported race was coded as American Indian or Alaska Native, Asian, Black or African American (hereafter, Black), White, and other race (including Native Hawaiian, Pacific Islander, >1 race, and “some other race”). Hispanic or Latino (hereafter, Hispanic) ethnicity was coded as Chicano, Mexican, or Mexican American (hereafter, Mexican); non-Hispanic or non-Latino (hereafter, non-Hispanic), and other Hispanic or Latino (including Central American, Cuban, Dominican, Puerto Rican, South American, and other Hispanic, Latino, or Spanish; hereafter, other Hispanic). We used White race and non-Hispanic ethnicity as reference groups for comparisons, conceptualizing differences as the result of structural, interpersonal, and internalized racism.

Frequency analyses were generated using SAS Survey Procedures software, version 9.4 (SAS Institute Inc). All proportion estimates in the text and tables were weighted to the US population using longitudinal weights supplied by the NCHS (eTable 1 in Supplement 1).

All rates per 1000 PY presented in the text and tables were both weighted to the US population and age standardized to reduce the effects of age differences when comparing subpopulations (eg, male vs female participants and those who did vs did not graduate college). Age standardizations were calculated using direct standardization to the 2010 US Census.^25,26^ The direct method estimates subpopulation rates using age-specific weights based on the age distribution of the 2010 US population.^25^

Relative risk (RR) was calculated using the PROC GENMOD package in SAS software, version 9.4, incorporating NHIS sampling characteristics while running multivariable Poisson regression models with robust SEs. Longitudinal sampling weights from the NHIS were used to produce data representative of the noninstitutionalized US civilian population 18 years and older. The threshold for statistical significance was 2-sided *P* = .05.

## Results

Among 10 415 individuals included in the analytic sample, 51.7% (95% CI, 50.3%-53.1%) were female, 48.3% (95% CI, 46.9%-49.7%) were male, 54.0% (95% CI, 52.4%-55.5%) were aged 18 to 49 years, 46.0% (95% CI, 44.5%-47.6%) were 50 years or older, 70.5% (95% CI, 69.1%-71.9%) were not college graduates, and 28.8% (95% CI, 27.4%-30.2%) were college graduates. Participants were followed up for a mean (SD) of 1.3 (0.3) years. With regard to race, 1.8% (95% CI, 1.0%-2.7%) of participants were American Indian or Alaska Native, 5.8% (95% CI, 4.9%-6.6%) were Asian, 12.2% (95% CI, 10.8%-13.6%) were Black, 72.6% (95% CI, 70.7%-74.6%) were White, 1.2% (95% CI, 0.9%-1.5%) were of other races, and 6.4% (95% CI, 5.4%-7.4%) were of unknown race. With regard to ethnicity, 16.5% (95% CI, 14.7%-18.4%) of participants were Hispanic. We found that those of Mexican ethnicity constituted 10.1% (95% CI, 8.5%-11.6%) of the sample, with 6.4% (95% CI, 5.3%-7.5%) being of other Hispanic ethnicity. Overall, 84.5% (95% CI, 81.6%-85.3%) of participants were non-Hispanic. There was excellent balance (ie, absolute standard difference <0.1) in the demographic distributions of 2019 NHIS participants by NHIS-LC participation status (Table 1). One exception noted was that individuals not enrolled in the NHIS-LC were more likely to have an unknown pain status. However, we found no evidence of differences between the 3 discrete pain categories of focus.

At baseline, 40.3% (95% CI, 38.8%-41.8%) of participants reported no pain, 38.9% (95% CI, 37.5%-40.2%) reported nonchronic pain, and 20.8% (95% CI, 19.6%-21.9%) reported chronic pain (Table 1). Most participants reported the same pain status at both their 2019 baseline report and their 2020 follow-up; for example, 62.3% (95% CI, 60.0%-64.4%) of those pain free at baseline remained pain free at follow-up, 54.0% (95% CI, 51.9%-56.2%) of those with nonchronic pain in 2019 continued to report nonchronic pain in 2020, and 61.4% (95% CI, 58.6%-64.1%) of those with chronic pain at baseline also reported chronic pain at follow-up (eTable 1 in Supplement 1). Among those pain free in 2019, the 1-year cumulative incidence for chronic pain in 2020 was 6.3% (95% CI, 5.3%-7.3%), while the 1-year cumulative incidence for HICP was 1.4% (95% CI, 0.9%-1.9%). We also observed evidence for both pain progression and pain recovery. Of those reporting nonchronic pain in 2019, 14.9% (95% CI, 13.5%-16.3%) had progressed to chronic pain at follow-up; of those reporting chronic pain in 2019, 10.4% (95% CI, 8.4%-12.3%) had fully recovered (ie, were pain free) in 2020.

We saw large differences in chronic pain rates by initial pain status. Among those without pain in 2019, the rate of incident chronic pain in 2020 was 52.4 (95% CI, 44.9-59.9) cases per 1000 PY, while the rate of incident HICP was 12.0 (95% CI, 8.2-15.8) cases per 1000 PY (Figure and Table 2). The rate of chronic pain in 2020 was substantially higher in those with baseline nonchronic pain (116.2 [95% CI, 105.3-127.1] cases per 1000 PY) (Table 3) and higher still in those with baseline chronic pain (462.0 [95% CI, 439.7-484.3] cases per 1000 PY) (Table 4). High rates of HICP in 2020 were seen in those with chronic pain at baseline (189.7 [95% CI, 149.4-230.1] cases per 1000 PY) and HICP at baseline (361.2 [95% CI, 265.6-456.8] cases per 1000 PY).

**Figure.**
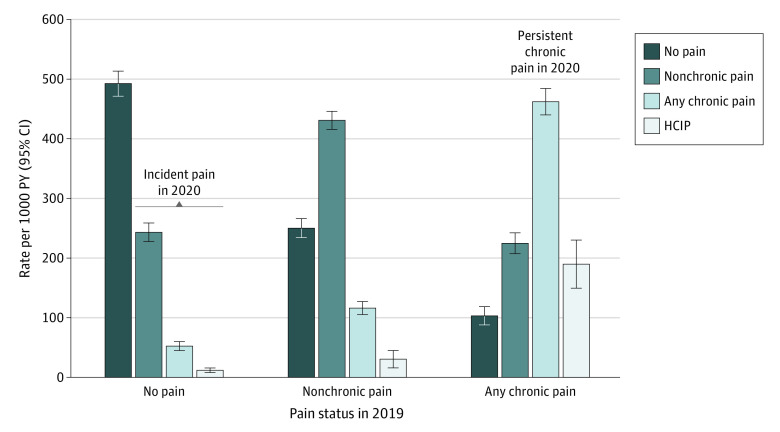
Rates of Pain in 2020 by Pain Status in 2019 No pain was defined as no pain in the past 3 months, nonchronic pain as pain on some days in the past 3 months, and chronic pain as pain on most days or every day in the past 3 months. High-impact chronic pain (HICP) was defined as chronic pain that limited life or work activities on most days or every day during the past 3 months. Rates were estimated using longitudinal survey weights supplied by the National Center for Health Statistics^23^ (10 415 participants included in the analysis; total weighted population of 250.9 million adults who were age standardized to the age distribution of the US population in 2010). The whiskers represent 95% CIs. PY indicates person-years.

**Table 2.** Number of Events, PY, Chronic Pain IRs, and RR of Chronic Pain in 2020 Among Adult Participants With No Reported Pain in 2019 but Chronic Pain in 2020

Characteristic	Raw No. of events	Weighted data				
		No. of events, thousands	PY, thousands	Age-standardized IR per 1000 PY (95% CI)	Unadjusted RR (95% CI)	Adjusted RR (95% CI)^a^
All adults	262	6352	126 526	52.4 (44.9-59.9)	NA	NA
Age range, y						
18-49	104	3519	82 541	42.6 (38.9-52.4)	1 [Reference]	1 [Reference]
≥50	158	2833	43 985	64.4 (52.4-76.4)	1.49 (1.10-2.02)	1.49 (1.11-2.02)
Sex						
Male	126	3248	64 335	53.0 (41.5-64.4)	1 [Reference]	1 [Reference]
Female	136	3103	62 192	51.7 (41.3-62.1)	0.97 (0.70-1.36)	0.99 (0.71-1.38)
Race						
American Indian or Alaska Native	7	231	1776	UR	NA	NA
Asian	9	387	10 625	UR	NA	NA
Black or African American	26	883	15 592	59.6 (31.0-88.2)	1.20 (0.70-2.06)	1.21 (0.71-2.05)
White	195	4160	87 071	50.0 (42.1-57.8)	1 [Reference]	1 [Reference]
Other^b^	5	89	1368	UR	NA	NA
Unknown	20	601	10 094	89.9 (60.9-118.9)	1.37 (0.54-2.50)	1.42 (0.71-2.86)
Hispanic or Latino ethnicity						
Chicano, Mexican, or Mexican American	22	587	15 224	44.5 (25.0-63.9)	1 [Reference]	1 [Reference]
Non-Hispanic or non-Latino	216	5014	100 294	52.7 (44.4-61.0)	1.27 (0.76-2.13)	1.43 (0.83-2.48)
Other Hispanic or Latino^c^	24	750	10 979	83.7 (58.7-108.8)	1.74 (0.76-2.13)	1.79 (0.96-3.34)
College attainment						
No degree	170	4653	86 870	57.5 (47.6-67.3)	1.26 (0.90-1.76)	1.22 (0.86-1.73)
Degree	90	1659	38 881	46.7 (35.6-57.9)	1 [Reference]	1 [Reference]
Unknown	2	39	775	UR	NA	NA

Abbreviations: IRs, incidence rates; NA, not applicable; PY, person-years; RR, relative risk; UR, unreliable (data did not meet National Center for Health Statistics standards for reliability in national surveys^24^).

^a^ Relative risks were adjusted for sex, age, race, Hispanic or Latino ethnicity, and college attainment.

^b^ Other race includes self-reported Native Hawaiian, Pacific Islander, more than 1 race, and “some other race.”

^c^ Other Hispanic or Latino ethnicity includes self-reported Central American, Cuban, Dominican, Puerto Rican, South American, and other Hispanic, Latino, or Spanish.

**Table 3.** Number of Events, PY, Chronic Pain IRs, and RR of Chronic Pain in 2020 Among Adult Participants With Nonchronic Pain in 2019 but Chronic Pain in 2020

Characteristic	Raw No. of events	Weighted data				
		No. of events, thousands	PY, thousands	Age-standardized IR per 1000 PY (95% CI)	Unadjusted RR (95% CI)	Adjusted RR (95% CI)^a^
All adults	676	14 551	121 499	116.2 (105.3-127.1)	NA	NA
Age range, y						
18-49	183	5854	62 397	93.8 (77.4-110.2)	1 [Reference]	1 [Reference]
≥50	493	8697	59 102	147.2 (133.7-160.6)	1.57 (1.29-1.92)	1.51 (1.24-1.84)
Sex						
Male	281	6308	57 056	107.9 (91.6-124.2)	1 [Reference]	1 [Reference]
Female	395	8242	64 442	123.2 (107.2-139.3)	1.14 (0.93-1.40)	1.14 (0.93-1.38)
Race						
American Indian or Alaska Native	9	363	2135	UR	NA	NA
Asian	15	284	6102	54.7 (31.8-77.5)	0.38 (0.21-0.69)	0.40 (0.22-0.72)
Black or African American	70	1929	15 363	130.4 (98.0-162.9)	1.06 (0.75-1.42)	0.99 (0.74-1.33)
White	554	10 720	88 693	114.5 (103.3-125.7)	1 [Reference]	1 [Reference]
Other^b^	3	145	1485	UR	NA	NA
Unknown	25	1108	7712	136.1 (62.4-209.9)	1.19 (0.73-1.94)	2.36 (1.61-4.80)
Hispanic or Latino ethnicity						
Chicano, Mexican, or Mexican American	25	1073	12 636	98.0 (58.6-137.4)	1 [Reference]	1 [Reference]
Non-Hispanic or non-Latino	629	12 760	102 762	120.3 (109.3-131.2)	1.45 (0.89-2.35)	2.28 (1.16-4.49)
Other Hispanic or Latino^c^	21	710	6038	128.9 (57.8-200.0)	1.35 (0.62-2.90)	1.41 (0.99-2.99)
College attainment						
No degree	421	10 895	81 339	132.1 (116.6-147.6)	1.50 (1.20-1.78)	1.40 (1.15-1.71)
Degree	252	3596	39 272	83.1 (70.5-95.7)	1 [Reference]	1 [Reference]
Unknown	3	59	888	UR	NA	NA

Abbreviations: IRs, incidence rates; NA, not applicable; PY, person-years; RR, relative risk; UR, unreliable (data did not meet National Center for Health Statistics standards for reliability in national surveys^24^).

^a^ Relative risks were adjusted for sex, age, race, Hispanic or Latino ethnicity, and college attainment.

^b^ Other race includes self-reported Native Hawaiian, Pacific Islander, more than 1 race, and “some other race.”

^c^ Other Hispanic or Latino ethnicity includes self-reported Central American, Cuban, Dominican, Puerto Rican, South American, and other Hispanic, Latino, or Spanish.

**Table 4.** Number of Events, PY, Chronic Pain IRs, and RR of Chronic Pain in 2020 Among Adult Participants With Chronic Pain in 2019 and 2020

Characteristic	Raw No. of events	Weighted data				
		No. of events, thousands	PY, thousands	Age-standardized IR per 1000 PY (95% CI)	Unadjusted RR (95% CI)	Adjusted RR (95% CI)^a^
All adults	1541	31 962	64 747	462.0 (439.7-484.3)	NA	NA
Age range, y						
18-49	314	9953	23 897	416.5 (379.5-453.5)	1 [Reference]	1 [Reference]
≥50	1227	22 009	40 850	538.8 (518.3-559.2)	1.30 (1.17-1.44)	1.29 (1.17-1.43)
Sex						
Male	623	14 066	29 738	448.7 (415.1-482.3)	1 [Reference]	1 [Reference]
Female	971	17 880	34 993	483.9 (464.2-503.6)	1.08 (0.99-1.18)	1.07 (0.98-1.16)
Unknown	1	16	16	UR	NA	NA
Race						
American Indian or Alaska Native	48	1030	1873	575.0 (544.4-605.5)	1.08 (0.90-1.32)	1.07 (0.87-1.32)
Asian	13	281	1306	267.6 (231.3-304.0)	0.42 (0.21-0.82)	0.41 (0.21-0.83)
Black or African American	141	3350	7077	433.5 (369.7-497.3)	0.95 (0.81-1.12)	0.94 (0.81-1.10)
White	1300	26 172	51 776	473.9 (450.7-497.1)	1 [Reference]	1 [Reference]
Other^b^	13	353	808	466.2 (263.0-669.5)	0.86 (0.55-1.20)	0.90 (0.53-1.53)
Unknown	26	1906	775	364.4 (157.6-571.3)	0.82 (0.55-1.20)	1.02 (0.66-1.60)
Hispanic or Latino ethnicity						
Chicano, Mexican, or Mexican American	44	1500	3890	383.1 (264.6-501.7)	1 [Reference]	1 [Reference]
Non-Hispanic or non-Latino	1446	29 205	57 936	426.0 (395.6-456.4)	1.33 (0.94-1.89)	1.33 (0.93-1.91)
Other Hispanic or Latino^c^	50	1236	2870	333.0 (204.5-461.5)	1.21 (0.78-1.85)	1.16 (0.77-1.74)
Unknown	1	20	51	UR	NA	NA
College attainment						
No degree	1137	26 488	52 223	479.5 (455.2-503.9)	1.16 (1.06-1.26)	1.20 (1.07-1.28)
Degree	398	5234	11 915	347.7 (304.8-390.7)	1 [Reference]	1 [Reference]
Unknown	6	240	609	UR	NA	NA

Abbreviations: IRs, incidence rates; NA, not applicable; PY, person-years; RR, relative risk; UR, unreliable (data did not meet National Center for Health Statistics standards for reliability in national surveys^24^).

^a^ Relative risks were adjusted for sex, age, race, Hispanic or Latino ancestry, and college attainment.

^b^ Other race includes self-reported Native Hawaiian, Pacific Islander, more than 1 race, and “some other race.”

^c^ Other Hispanic or Latino ethnicity includes self-reported Central American, Cuban, Dominican, Puerto Rican, South American, and other Hispanic, Latino, or Spanish.

These large differences in chronic pain rates were present across several demographic categories (Tables 2, 3, and 4). Regardless of baseline pain status, older participants had higher rates of chronic pain than younger participants, and those without a college degree had higher rates of chronic pain than those with a college degree. Asian participants with nonchronic or chronic pain at baseline had lower rates of chronic pain compared with White participants (Tables 3 and 4). Regardless of baseline pain status, no differences in the rates of chronic pain were seen between male and female participants. Non-Hispanic participants with nonchronic pain at baseline had higher rates of chronic pain in 2020 than Mexican participants (Table 3). Otherwise, no differences in chronic pain rates were noted based on Hispanic ethnicity.

All of the aforementioned differences in rates were confirmed in adjusted regression analyses. For instance, regardless of baseline pain status, those 50 years or older had a higher risk of chronic pain compared with those aged 18 to 49 years (no reported pain at baseline: adjusted RR [ARR], 1.49 [95% CI, 1.11-2.02]; nonchronic pain at baseline: ARR, 1.51 [95% CI, 1.24-1.84]; and chronic pain at baseline: ARR, 1.29 [95% CI, 1.17-1.43]). Among those with baseline nonchronic or chronic pain, those without a college degree had a higher risk of chronic pain compared with those with a college degree (nonchronic pain at baseline: ARR, 1.40 [95% CI, 1.15-1.71]; chronic pain at baseline: ARR, 1.20 [95% CI, 1.07-1.28]).

## Discussion

In this cohort study, nearly two-thirds (61.4%) of adults with chronic pain in 2019 continued to have chronic pain in 2020. While 14.9% of those with nonchronic pain reported chronic pain 1 year later, only 6.3% of those pain free in 2019 developed incident chronic pain and only 1.4% exhibited an onset of HICP. Lower educational attainment and older age were associated with higher rates of chronic pain in 2020 regardless of pain status in 2019. Of note, the incidence of chronic pain (52.4 cases per 1000 PY) was high compared with other chronic diseases and conditions for which the incidence in the US adult population is known, including diabetes (7.1 cases per 1000 PY),^27^ depression (15.9 cases per 1000 PY),^28^ and hypertension (45.3 cases per 1000 PY).^29^

Although chronic pain is sometimes assumed to persist indefinitely, our finding that 10.4% of adults with chronic pain experienced improvement over time is consistent with previous evidence from studies in Denmark,^30^ Norway,^31^ Sweden,^32^ and the UK,^33^ which revealed rates ranging from 5.4% to 8.7% (eTable 2 in Supplement 1). Also similar across our study and these 4 studies^30,31,32,33^ were the rates of 1-year cumulative incidence for chronic pain at baseline, which ranged from 1.8%^30^ to 8.3%^32^ (eTable 2 in Supplement 1). The observed differences likely reflect variability in study methods, including how chronic pain was defined, the populations studied, and the length of follow-up. The rates for persistent chronic pain varied from 47.9%^30^ in the youngest cohort (aged ≥16 years at entry) to 93.5%^32^ in the oldest cohort (aged ≥65 years at entry). These rates suggest an age effect consistent with our finding that participants 50 years or older had a 29% higher adjusted RR of persistent pain than younger participants. Our ongoing research examines the underlying factors that may explain the observed differences in chronic pain incidence, persistence, and recovery rates in our study.

### Limitations

This study has several limitations. Despite the strengths and generalizability of our population-based analysis, there are also limitations inherent to the data. First, the NHIS-LC does not collect information on the underlying causes for the reported pain, and data are collected only twice across 2 years of follow-up, rendering a detailed trajectory of pain difficult to assess. Second, there remains the possibility that those experiencing new or persistent chronic pain or HICP were less likely to participate in the 2020 follow-up survey, which could lead to an underestimation of rates. In turn, reduced statistical power could obscure real differences in our subpopulations. Third, although the longitudinal cohort contained a large sample and the NHIS oversamples some racial and ethnic minority groups, the small samples of American Indian or Alaska Native and Asian participants precluded rate calculations for incident chronic pain. Similarly, we were limited to assessing dichotomous measures of age and educational attainment. Analyses of a substantially larger cohort might have revealed more nuanced differences by age and educational attainment. Fourth, while we were able to present overall data on the rates of HICP, the relatively small number of individuals with HICP prevented subgroup analyses.

## Conclusions

In this cohort study, the incidence of chronic pain (52.4 cases per 1000 PY) was high compared with other chronic diseases and conditions for which the incidence in the US adult population is known, including diabetes,^27^ depression,^28^ and hypertension.^29^ This comparison emphasizes the high disease burden of chronic pain in the US adult population and the need for both prevention and early management of pain before it can become chronic,^1,2^ especially for groups at higher risk.

## Notes

Supplement 1. **eTable 1.** Pain Status in 2020 by Pain Status in 2019 Among the National Health Interview Survey 2019-2020 Longitudinal Cohort **eTable 2.** Data Across Selected Chronic Pain Incidence Studies **eFigure.** Study Sample Flowchart
eReferences

Supplement 2.
Data Sharing Statement

## References

[1] Institute of Medicine (US) Committee on Advancing Pain Research, Care, and Education. Relieving Pain in America: A Blueprint for Transforming Prevention, Care, Education, and Research. National Academies Press (US); 2011. 22553896

[2] Interagency Pain Research Coordinating Committee. *National Pain Strategy: A Comprehensive Population Health–Level Strategy for Pain*. National Institutes of Health, US Dept of Health and Human Services; 2016. Accessed August 3, 2022. https://www.in.gov/health/overdose-prevention/files/NIH-National-Pain-Strategy.pdf

[3] Kennedy J, Roll JM, Schraudner T, Murphy S, McPherson S. Prevalence of persistent pain in the U.S. adult population: new data from the 2010 National Health Interview Survey. J Pain. 2014;15(10):979-984. doi:10.1016/j.jpain.2014.05.009 25267013

[4] Nahin RL. Estimates of pain prevalence and severity in adults: United States, 2012. J Pain. 2015;16(8):769-780. doi:10.1016/j.jpain.2015.05.002 26028573 PMC4562413

[5] Nahin RL. Pain prevalence, chronicity and impact within subpopulations based on both Hispanic ancestry and race: United States, 2010-2017. J Pain. 2021;22(7):826-851. doi:10.1016/j.jpain.2021.02.006 33636375

[6] Pitcher MH, Von Korff M, Bushnell MC, Porter L. Prevalence and profile of high-impact chronic pain in the United States. J Pain. 2019;20(2):146-160. doi:10.1016/j.jpain.2018.07.006 30096445 PMC8822465

[7] Zelaya CE, Dahlhamer JM, Lucas JW, Connor EM. Chronic pain and high-impact chronic pain among U.S. adults, 2019. NCHS Data Brief. 2020;(390):1-8. 33151145

[8] Felson DT, Zhang Y, Hannan MT, . The incidence and natural history of knee osteoarthritis in the elderly: the Framingham Osteoarthritis Study. Arthritis Rheum. 1995;38(10):1500-1505. doi:10.1002/art.1780381017 7575700

[9] Jarvik JG, Hollingworth W, Heagerty PJ, Haynor DR, Boyko EJ, Deyo RA. Three-year incidence of low back pain in an initially asymptomatic cohort: clinical and imaging risk factors. Spine (Phila Pa 1976). 2005;30(13):1541-1548. doi:10.1097/01.brs.0000167536.60002.87 15990670

[10] Katusic S, Beard CM, Bergstralh E, Kurland LT. Incidence and clinical features of trigeminal neuralgia, Rochester, Minnesota, 1945-1984. Ann Neurol. 1990;27(1):89-95. doi:10.1002/ana.410270114 2301931

[11] Moss AS, Murphy LB, Helmick CG, . Annual incidence rates of hip symptoms and three hip OA outcomes from a U.S. population-based cohort study: the Johnston County Osteoarthritis Project. Osteoarthritis Cartilage. 2016;24(9):1518-1527. doi:10.1016/j.joca.2016.04.012 27109873 PMC5466003

[12] Murphy LB, Moss S, Do BT, . Annual incidence of knee symptoms and four knee osteoarthritis outcomes in the Johnston County Osteoarthritis Project. Arthritis Care Res (Hoboken). 2016;68(1):55-65. doi:10.1002/acr.22641 26097226 PMC4684807

[13] Scher AI, Stewart WF, Ricci JA, Lipton RB. Factors associated with the onset and remission of chronic daily headache in a population-based study. Pain. 2003;106(1-2):81-89. doi:10.1016/S0304-3959(03)00293-8 14581114

[14] Von Korff M, Resche LL, Dworkin SF. First onset of common pain symptoms: a prospective study of depression as a risk factor. Pain. 1993;55(2):251-258. doi:10.1016/0304-3959(93)90154-H 8309712

[15] Shahidi B, Curran-Everett D, Maluf KS. Psychosocial, physical, and neurophysiological risk factors for chronic neck pain: a prospective inception cohort study. J Pain. 2015;16(12):1288-1299. doi:10.1016/j.jpain.2015.09.002 26400680 PMC9288140

[16] Waterman BR, Belmont PJ Jr, Schoenfeld AJ. Low back pain in the United States: incidence and risk factors for presentation in the emergency setting. Spine J. 2012;12(1):63-70. doi:10.1016/j.spinee.2011.09.002 21978519

[17] Singh JA, Cleveland JD. Gout and chronic pain in older adults: a Medicare claims study. Clin Rheumatol. 2019;38(7):1953-1960. doi:10.1007/s10067-019-04526-0 30927116

[18] Schmaling KB, Nounou ZA. Incident chronic spinal pain and depressive disorders: data from the National Comorbidity Survey. J Pain. 2019;20(4):481-488. doi:10.1016/j.jpain.2018.11.002 30471429 PMC6433529

[19] Crane MM, Juneja M, Allen J, . Epidemiology and treatment of new-onset and established rheumatoid arthritis in an insured US population. Arthritis Care Res (Hoboken). 2015;67(12):1646-1655. doi:10.1002/acr.22646 26097059

[20] Magni G, Marchetti M, Moreschi C, Merskey H, Luchini SR. Chronic musculoskeletal pain and depressive symptoms in the National Health and Nutrition Examination. I. epidemiologic follow-up study. Pain. 1993;53(2):163-168. doi:10.1016/0304-3959(93)90076-2 8336986

[21] Centers for Disease Control and Prevention. *Proposed Objectives for Inclusion in Healthy People 2030*. US Dept of Health and Human Services; 2020. Accessed August 3, 2022. https://wayback.archive-it.org/5774/20190104071714/https://www.healthypeople.gov/sites/default/files/ObjectivesPublicComment508.pdf

[22] Von Elm E, Altman DG, Egger M, Pocock SJ, Gøtzsche PC, Vandenbroucke JP; STROBE Initiative. The Strengthening the Reporting of Observational Studies in Epidemiology (STROBE) statement: guidelines for reporting observational studies. Lancet. 2007;370(9596):1453-1457. doi:10.1016/S0140-6736(07)61602-X 18064739

[23] Division of Health Interview Statistics, National Center for Health Statistics. *National Health Interview Survey: 2020 Survey Description*. Centers for Disease Control and Prevention, US Dept of Health and Human Services; 2021. Accessed August 3, 2022. https://ftp.cdc.gov/pub/Health_Statistics/NCHS/Dataset_Documentation/NHIS/2020/srvydesc-508.pdf

[24] Parker JD, Talih M, Malec DJ, . National Center for Health Statistics data presentation standards for proportions. Vital Health Stat 2. 2017;175(175):1-22. 30248016

[25] Howden LM, Meyer JA. Age and sex composition: 2010. United States Census Bureau. May 1, 2011. Accessed April 18, 2023. https://www.census.gov/content/dam/Census/library/publications/2011/dec/c2010br-03.pdf

[26] Li C, Ford ES, Zhao G, Wen XJ, Gotway CA. Age adjustment of diabetes prevalence: use of 2010 U.S. Census data. J Diabetes. 2014;6(5):451-461. doi:10.1111/1753-0407.12122 24393518 PMC11287713

[27] Geiss LS, Wang J, Cheng YJ, . Prevalence and incidence trends for diagnosed diabetes among adults aged 20 to 79 years, United States, 1980-2012. JAMA. 2014;312(12):1218-1226. doi:10.1001/jama.2014.11494 25247518

[28] Eaton WW, Kramer M, Anthony JC, Dryman A, Shapiro S, Locke BZ. The incidence of specific *DIS*/*DSM-III* mental disorders: data from the NIMH Epidemiologic Catchment Area Program. Acta Psychiatr Scand. 1989;79(2):163-178. doi:10.1111/j.1600-0447.1989.tb08584.x 2784251

[29] Neufcourt L, Zins M, Berkman LF, Grimaud O. Socioeconomic disparities and risk of hypertension among older Americans: the Health and Retirement Study. J Hypertens. 2021;39(12):2497-2505. doi:10.1097/HJH.0000000000002959 34387572

[30] Eriksen J, Ekholm O, Sjøgren P, Rasmussen NK. Development of and recovery from long-term pain: a 6-year follow-up study of a cross-section of the adult Danish population. Pain. 2004;108(1-2):154-162. doi:10.1016/j.pain.2003.12.018 15109519

[31] Landmark T, Dale O, Romundstad P, Woodhouse A, Kaasa S, Borchgrevink PC. Development and course of chronic pain over 4 years in the general population: the HUNT pain study. Eur J Pain. 2018;22(9):1606-1616. doi:10.1002/ejp.1243 29754398

[32] Larsson C, Hansson EE, Sundquist K, Jakobsson U. Chronic pain in older adults: prevalence, incidence, and risk factors. Scand J Rheumatol. 2017;46(4):317-325. doi:10.1080/03009742.2016.1218543 27885914

[33] Elliott AM, Smith BH, Hannaford PC, Smith WC, Chambers WA. The course of chronic pain in the community: results of a 4-year follow-up study. Pain. 2002;99(1-2):299-307. doi:10.1016/S0304-3959(02)00138-0 12237208

