# Supplementary material for: Estimated Rates of Incident and Persistent Chronic Pain Among US Adults, 2019-2020
Source: JAMA Netw Open. 2023 May 16;6(5):e2313563. doi: 10.1001/jamanetworkopen.2023.13563 (PMC10189566; doi:10.1001/jamanetworkopen.2023.13563)
Supplement: Supplement 1. — eTable 1. Pain Status in 2020 by Pain Status in 2019 Among the National Health Interview Survey 2019-2020 Longitudinal Cohort eTable 2. Data Across Selected Chronic Pain Incidence Studies eFigure. Study Sample Flowchart eReferences [file jamanetwopen-e2313563-s001.pdf]

Supplementary Online Content

Nahin RL, Feinberg T, Kapos FP, Terman GW. Estimated rates of incident and persistent chronic pain among US adults, 2019-2020. *JAMA Netw Open*. 2023;6(5):e2313563. doi:10.1001/jamanetworkopen.2023.13563

eTable 1. Pain Status in 2020 by Pain Status in 2019 Among the National Health Interview Survey 2019-2020 Longitudinal Cohort

eTable 2. Data Across Selected Chronic Pain Incidence Studies

eFigure. Study Sample Flowchart

eReferences

This supplemental material has been provided by the authors to give readers additional information about their work.

© 2023 Nahin RL et al. *JAMA Network Open*.

eTable 1. Pain Status in 2020 by Pain Status in 2019 Among the National Health Interview Survey 2019-2020 Longitudinal Cohort

2019 Pain Status	2020 Pain Status	Raw Frequency	Weighted Frequency (1000's)	Weighted Percent	Lower 95% Confidence Limit	Upper 95% Confidence Limit
**No pain in the last 3 months**						
	No pain in the last 3 months	2293	63,037	62.3	60.0	64.6
	Non-chronic pain[a]	1168	30,277	29.9	27.8	32.0
	Chronic pain[b]	262	6,352	6.3	5.3	7.3
	High-impact chronic pain[c]	63	1,413	1.4	0.9	1.9
	Unknown pain status	53	1,478	1.5	0.9	2.0
	Total[d]	3776	101,143	100.0		
**Non-chronic pain[a]**						
	No pain in the last 3 months	1121	29,041	29.8	27.7	31.9
	Non-chronic pain[a]	2335	52,695	54.0	51.9	56.2
	Chronic pain[b]	676	1,451	14.9	13.5	16.3
	High-impact chronic pain[c]	157	3,762	3.9	3.0	4.7
	Unknown pain status	53	1,220	1.3	0.8	1.7
	Total[d]	4185	97,507	100.0		

© 2023 Nahin RL et al. *JAMA Network Open*.

2019 Pain Status	2020 Pain Status	Raw Frequency	Weighted Frequency (1000's)	Weighted Percent	Lower 95% Confidence Limit	Upper 95% Confidence Limit
**Chronic pain[b]**						
	No pain in the last 3 months	210	5,405	10.4	8.4	12.3
	Non-chronic pain[a]	661	13,876	26.6	24.2	29.1
	Chronic pain[b]	1,541	31,962	61.4	58.6	64.1
	High-impact chronic pain[c]	676	13,957	26.8	24.2	29.4
	Unknown pain status	34	837	1.6	0.7	2.5
	Total[d]	2,446	52,080	100.0		
**Total**						
	No pain in the last 3 months	3,625	97,586	38.9	37.3	40.4
	Non-chronic pain[a]	4,167	96,870	38.6	37.3	39.9
	Chronic pain[b]	2,482	52,918	21.1	20.0	22.2
	High-impact chronic pain[c]	896	19,133	7.6	6.9	8.3
	Unknown pain status	141	3,543	1.4	1.1	1.7
	Total[d]	10,415	250,917	100.0		

Footnotes:
a: Pain ‘some days’ in the last 3 months
b: Pain ‘most days’ or ‘every day’ in the last 3 months
c: Chronic pain that limited life or work activities ‘most days’ or every day’ in the last 3 months
d: Denominator based on the sum of No pain, Non-chronic pain, Chronic pain and Unknown pain status. High-impact chronic pain is a subgroup of Chronic pain and does not add independently to the denominator

© 2023 Nahin RL et al. *JAMA Network Open*.

eTable 2. Data Across Selected Chronic Pain Incidence Studies

Lead Author	Eriksen et al[1]	Landmark et al[2]	Larsson et al[3]	Elliott et al[4]	Nahin et al[5]
**Country**	Denmark	Norway	Sweden	United Kingdom	United States
**Construction of Cohort**	National random sample of those 16+	Regional random sample of those 20+	National random sample of those 65+	Regional random sample of those 25+	National random sample of those 18+
**Sample Size at follow-up**	2,649	3,405	843	1,608	10,415
**Years of follow-up**	6	1	1	4	1.3 (average)
	Annualized proportions				
**No pain ===> No pain**	89.7%[a]	84.2%	Unknown	66.7%	62.3%
**Cumulative Incident chronic pain No pain ===> Chronic pain**	1.8%[a]	4.8%	5.4%	8.3%	6.3%
**Persistent chronic pain Chronic pain ===> Chronic pain**	47.9%[a]	70.8%	93.5%	78.5%	61.4%
**Recovery from chronic pain Chronic pain ===> No pain**	8.7%[a]	8.4%	Unknown	5.4%	10.4%

Footnote:
a = hand calculated based on data presented in Table 2 of Eriksen et al[1]

© 2023 Nahin RL et al. *JAMA Network Open*.

eFigure. Study Sample Flowchart

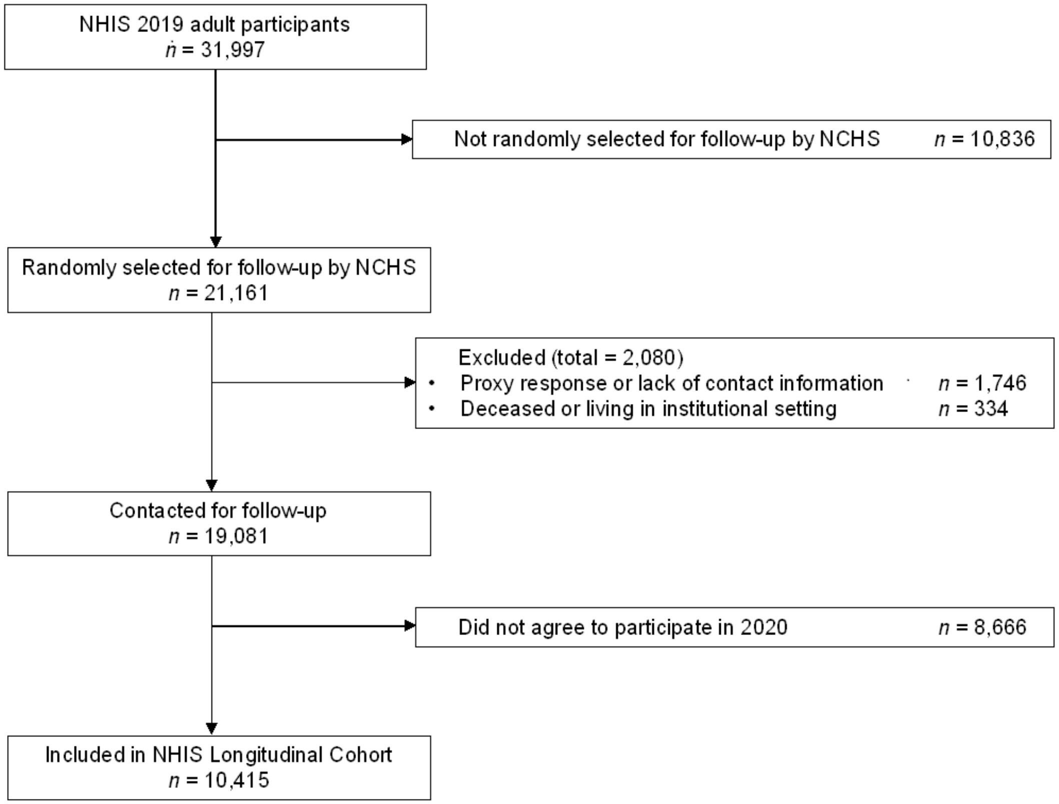

NHIS: National Health Interview Survey
NCHS: National Center for Health Statistics

© 2023 Nahin RL et al. *JAMA Network Open*.

eReferences

1. Eriksen J, Ekholm O, Sjøgren P, Rasmussen NK. Development of and recovery from long-term pain. A 6-year follow-up study of a cross-section of the adult Danish population. Pain. 2004 Mar;108(1-2):154-62.
2. Landmark T, Dale O, Romundstad P, Woodhouse A, Kaasa S, Borchgrevink PC. Development and course of chronic pain over 4 years in the general population: The HUNT pain study. Eur J Pain. 2018 Oct;22(9):1606-1616.
3. Larsson C, Hansson EE, Sundquist K, Jakobsson U. Chronic pain in older adults: prevalence, incidence, and risk factors. Scand J Rheumatol. 2017 Jul;46(4):317-325.
4. Elliott AM, Smith BH, Hannaford PC, Smith WC, Chambers WA. The course of chronic pain in the community: results of a 4-year follow-up study. Pain. 2002 99(1-2):299-307.
5. Nahin RL, Feinberg T, Kapos FP, Terman GW. Estimated rates of Incident and Persistent Chronic Pain Rates in US adults: 2019-2020; JAMA Open Network, 2023.

© 2023 Nahin RL et al. *JAMA Network Open*.
