# Supplementary material for: Estimated Rates of Incident and Persistent Chronic Pain Among US Adults, 2019-2020
Source: JAMA Netw Open. 2023 May 16;6(5):e2313563. doi: 10.1001/jamanetworkopen.2023.13563 (PMC10189566; doi:10.1001/jamanetworkopen.2023.13563)
Supplement: Supplement 2. — Data Sharing Statement [file jamanetwopen-e2313563-s002.pdf]

# Data Sharing Statement

Nahin. Estimated Rates of Incident and Persistent Chronic Pain Among US Adults, 2019-2020. *JAMA Netw Open*. Published May 16, 2023. doi:10.1001/jamanetworkopen.2023.13563

**Data**
**Data available:** Yes
**Data types:** Deidentified participant data
**How to access data:** https://www.cdc.gov/nchs/nhis/data-questionnaires-documentation.htm
**When available:** beginning date: 09-01-2021

**Supporting Documents**
**Document types:** None

**Additional Information**
**Who can access the data:** All publicly available National Health Interview Survey data are available to anyone with internet access
**Types of analyses:** The data are currently available
**Mechanisms of data availability:** Any individual may access the data posted on the CDC/NCHS/NHIS website listed above
